# Epidemic *Clostridium difficile* Strain in Hospital Visitation Dog

**DOI:** 10.3201/eid1206.060115

**Published:** 2006-06

**Authors:** Sandra L. Lefebvre, Luis G. Arroyo, J. Scott Weese

**Affiliations:** * University of Guelph, Guelph, Ontario, Canada

**Keywords:** Clostridium difficile, dog, zoonoses, letter

**To the Editor:** Rates of illness and death from *Clostridium difficile*–associated disease (CDAD) and reports of CDAD in persons without traditional risk factors (*1*) have been increasing. One particular strain of *C. difficile* has been implicated in outbreaks of CDAD in hospitals in North America and Europe and appears to be spreading internationally at an alarming rate. This strain is classified as ribotype 027, toxinotype III, and possesses genes encoding toxins A, B, and CDT (binary toxin) as well as a deletion in the *tcdC* gene, which is believed to increase virulence (*2*).

We report this toxin-variant strain of *C. difficile* in a healthy, 4-year-old toy poodle that visits persons in hospitals and long-term care facilities in Ontario on a weekly basis. *C. difficile* was isolated from a fecal sample collected in the summer of 2004 as part of a cross-sectional study evaluating pathogen carriage by visitation dogs (*3*). The isolate was subsequently characterized by ribotyping (*4*) and by polymerase chain reaction (PCR) detection of genes that encode production of toxins A and B (*5*). Toxin CDT was confirmed by amplifying the portion of the gene (*cdtB*) that encodes for the receptor-binding component of the toxin, according to a previously reported protocol (*6*). As a result, the isolate was classified as ribotype 027, toxinotype III (*7*), and was found to possess all 3 toxin genes. The *tcdC* gene deletion was also confirmed with PCR (*8*).

These results indicate that this canine isolate is indistinguishable from the major strain implicated in outbreaks of highly virulent CDAD around the world. According to the infection control practitioner at the hospital the dog visited, CDAD cases were occurring at increased frequency in the facility around the time the dog’s fecal specimen was collected. However, patient diagnosis was made solely through fecal toxin testing, and strains were not characterized. The facility has reported only sporadic cases of CDAD in the past few years.

This is the first report of this human, epidemic strain of *C. difficile* in a dog. Many *C. difficile* strains isolated from animals, including dogs, are indistinguishable from strains associated with disease in humans (*9*). To date, no study, including this one, has shown that interspecies transmission occurs; however, that possibility exists, as is becoming apparent with other pathogens, such as methicillin-resistant *Staphylococcus aureus*. The recurrent exposure of this dog to human healthcare settings suggests that the animal acquired this strain during visits to the hospital or long-term care facility, either from the healthcare environment or contaminated hands of human contacts. We recommend that future studies evaluating the dissemination of this strain and investigations of the movement of *C. difficile* into the community consider the role of animals.
